# Altered levels of salivary and plasma pain related markers in temporomandibular disorders

**DOI:** 10.1186/s10194-020-01160-z

**Published:** 2020-08-26

**Authors:** Hajer Jasim, Bijar Ghafouri, Björn Gerdle, Britt Hedenberg-Magnusson, Malin Ernberg

**Affiliations:** 1 Division of Oral Diagnostics & Rehabilitation, Department of Dental Medicine, Section for Orofacial Pain and Jaw function, Karolinska Institutet and Scandinavian Center for Orofacial neuroscience (SCON), BOX 4064, SE141 04 Huddinge, Sweden; 2 Pain and Rehabilitation Centre, and Department of Health, Medicine and Caring Sciences, Linköping University, SE581 83 Linköping, Sweden; 3 Folktandvården Stockholms Län AB, SE 11382 Stockholm, Sweden

**Keywords:** Biomarker; chronic pain; temporomandibular disorders, Saliva, Myalgia, NGF, BDNF, Glutamate, Substance P, 5-HT

## Abstract

**Background:**

Different pain syndromes may be characterized by different profiles of mediators reflecting pathophysiological differences, and these alterations may be measured in a simple saliva sample. The aims of the current study were to compare concentration of glutamate, serotonin (5-HT), nerve growth factor (NGF), brain-derived neurotrophic factor (BDNF), and substance P (SP) in saliva and plasma from a well-defined group of patients with chronic temporomandibular disorders myalgia (TMD-myalgia) with a group of pain-free controls, and further investigate the relationship between these markers and clinical characteristics.

**Methods:**

Patients diagnosed according to the diagnostic criteria for TMD (*n* = 39), and matched healthy pain-free controls (n = 39) were included. Stimulated whole saliva and plasma samples were collected in the morning. Glutamate was analysed using a colorimetric assay, and 5-HT and SP were analysed by commercially available ELISA. Levels of NGF and BDNF were determined using multiplex electrochemiluminescence assay panel.

**Results:**

Patients expressed higher salivary and plasma levels of glutamate (saliva: 40.22 ± 13.23 μmol/L; plasma: 30.31 ± 18.73 μmol/L) than controls (saliva: 33.24 ± 11.27 μmol/L; plasma: 20.41 ± 15.96 μmol/L) (*p* < 0.05). Salivary NGF (0.319 ± 0.261 pg/ml) and BDNF (3.57 ± 1.47 pg/ml) were lower in patients compared to controls (NGF: 0.528 ± 0.477 pg/ml; BDNF 4.62 ± 2.51 pg/ml)(p’s < 0.05). Contrary, plasma BDNF, was higher in patients (263.33 ± 245.13 pg/ml) than controls (151.81 ± 125.90 pg/ml) (*p* < 0.05). 5-HT was undetectable in saliva. Neither plasma 5-HT, nor SP levels differed between groups. BDNF and NGF concentrations correlated to levels of psychological distress (*p* < 0.0005).

**Conclusion:**

The higher levels of salivary and plasma glutamate in patients with TMD-myalgia compared to controls strengthens its importance in the pathophysiology of TMD-myalgia. However, the lack of correlation to pain levels question its role as a putative biomarker. Patients with TMD-myalgia further had lower levels of salivary NGF and BDNF, but higher plasma BDNF. These results and their correlations to psychological distress warrant further investigations.

## Background

Chronic masticatory muscle pain, i.e. temporomandibular disorder (TMD) myalgia, affects approximately 10% of the adult population, and is three times more frequent in women [1, 2]. It is characterized by spontaneous pain that is intensified by function, hyperalgesia and pain referral. TMD-myalgia is usually associated with comorbidities, such as depression, stress and other psychological factors [1–4].

There are several risk factors underlying TMD-myalgia, e.g. biophysiological, psychosocial, structural, postural and genetic factors [1, 5]. Despite extensive research, the nociceptive mechanisms underlying the pain are still mainly unknown [6]. However, there is agreement among scientists that both peripheral and central mechanisms contribute to the development and maintenance of the disorder. The role of central mechanisms seems to be more evident the longer the pain persists, but peripheral inputs are believed to drive the pain [5, 7]. There is increasing evidence for neurogenic derived inflammation in TMD-myalgia [6, 8]. These mechanisms involve release of neuropeptides by activation of peripheral sensory afferent neurons. These neuropeptides promotes in turn the release of other chemicals that continues to activate and sensitize neurons [6].

Glutamate is the main excitatory neurotransmitter in the nervous system and is presented both in central and peripheral nerve endings. It has been demonstrated that glutamate is released peripherally and centrally in response to nociceptive stimulation and tissue or nerve injury [9–12]. Serotonin (5-HT) is another molecule released in the periphery in response to tissue trauma and inflammation that has a role in activating and sensitizing peripheral neurons. It also sensitize afferent neurons to other substances such as glutamate and substance P (SP) [6]. Our research group has previously shown that intramuscular levels of glutamate and 5-HT are elevated in patients with TMD-myalgia [10, 13]. These increased levels of 5-HT also correlated with pain intensity and allodynia, and both mediators induce pain and hyperalgesia when injected into jaw muscles [13, 14]. Nerve growth factor (NGF) is a neuropeptide that modulates the expression of peripheral and central pain-related markers but also can sensitize adjacent nociceptive neurons in response to inflammation. Injection of NGF into the jaw muscles causes prolonged hyperalgesia [15, 16]. Furthermore, several studies have shown elevated levels of NGF in saliva, general circulation as well as locally in the synovial fluid of patients with different pain conditions [17–21]. There is only two studies that have explored salivary NGF in chronic pain; Jang and co-authors investigated saliva NGF in chronic migraine compared to healthy controls and found that patients exhibited higher levels of NGF, both in saliva and plasma [17]. Borelli and co-authors reported similar findings in burning mouth syndrome (BMS) [18].

Brain derived neurotropic factor (BDNF) and SP are other examples of neuropeptides that play significant roles in the development of pain and hyperalgesia [7, 22, 23]. BDNF have been implicated in the pathophysiology of BMS, migraine and other primary headaches based on its increased saliva and plasma concentration during active pain periods [17, 24–26]. There is also evidence that salivary SP levels increase with noxious stimulation, indicating that SP may play a role in central sensitization associated with chronic pain [17, 27]. These findings taken together indicate that ongoing activity in sensory neurons may be reflected in peripheral change of neuropeptide and neurotransmitter levels.

Most of the past studies on putative biomarkers of chronic pain have assessed plasma, cerebrospinal or interstitial muscle concentrations [6]. Relatively fewer studies have investigated biomarker levels in saliva in chronic pain [12, 17, 18, 22, 25, 26, 28–31]. Saliva can potentially be used as a specimen for diagnosis in TMD because it can exchange substances with blood. A thin layer of epithelial cells separating the salivary ducts from the systemic circulation enables the transfer of substances from the saliva by means of active carriage, diffusion through cell membrane, or passive diffusion [32]. Nevertheless, there are also disease-specific biomarkers that are only present in saliva but not in blood, including some biomarkers for oral cancer [33]. One may speculate that this could also be the case for some putative biomarkers of TMD myalgia because of the close vicinity between the jaw muscles and salivary glands.

Therefore, saliva can reflect the physiological state of the body and reveal systemic as well as local conditions in the glands and surrounding structures. Saliva collection provides several advantages over blood [34], such as being easy, non-invasive and cost-effective. There are therefore compelling reasons for exploring saliva as a diagnostic and prognostic fluid in TMD-myalgia [35, 36].

With the growing interested in the search of biomarkers reflecting chronic pain pathophysiology, it could be hypothesized that TMD-myalgia is mirrored in altered saliva levels of certain neuropeptides and other pain-related mediators. The main aim of this study was therefore to compare the concentration of glutamate, 5-HT, NGF, BDNF, and SP in saliva and plasma from a well-defined group of TMD-myalgia patients with a matched healthy pain free control group. An additional aim was to investigate the relationships between these markers and pain ratings and psychological factors, as well as between saliva and plasma concentration of these mediators.

## Methods

### Participants

Thirty-nine consecutive patients (mean + SD age: 28.8 ± 7.4 years) referred to the Specialist Clinic for Orofacial Pain and Jaw function, University Dental Clinic, Karolinska Institutet, Huddinge, Sweden were included in the study. Inclusion criteria were a diagnosis of myalgia or myofascial pain (TMD-myalgia) according to the Diagnostic Criteria for TMD (DC/TMD) axis I with at least three months duration and an average pain intensity during the last 30 days of ≥ 3/10 on a numeric rating scale (NRS).

Thirty-nine sex and age matched healthy controls (mean + SD age: 28.8 ± 6.9 years) were also included in the study. They had no current pain and were recruited though advertisement and among undergraduate dental students at Karolinska Institutet, Huddinge, Sweden.

A power calculation showed that 37 participants in each group should be enough to detect a difference in biomarker level of 1.5 SD with 80% power and a significant level of 5%.

Exclusion criteria for both groups were any conditions that could influence pain sensitivity, such as chronic widespread pain (e.g., fibromyalgia), systemic inflammatory disease (e.g., rheumatoid arthritis, ankylosing spondylitis, psoriatic arthritis), whiplash-associated disorder, neurological disorders, pain of dental origin, pregnancy or lactation, and high blood pressure. Medications that could interfere with analysis and pain sensitivity such as anticoagulant treatment and analgesic drugs, or that could interfere with pain perception such as antidepressants or anticonvulsant drugs were also considered exclusion criteria. Patients with factors that could influence saliva collection and composition, such as hypo-salivation, salivary gland diseases, poor oral hygiene, regular tobacco usage, several missing teeth, extensive prosthodontics rehabilitations, oral diseases and mucosal lesions were further excluded from further involvement in the study. All exclusions criteria were evaluated in each participant by taking medical history and through dental examination. One dentist (HJ) calibrated to a reference standard researcher (ME) according to the most recent DC/TMD criteria examined all the patients and controls to ensure they fulfilled all the terms.

### Clinical examination and questionnaires

All participants underwent a general clinical dental examination and were evaluated by the Swedish version of the DC/TMD axis I and II [37]. During the clinical examination, participants were checked for pronounced attrition, decayed teeth, periodontal diseases, mucosal lesions, oral hygiene as well occlusal contacts. Validated instruments included in the DC/TMD questionnaire were used to measure pain related physical functioning, symptoms of depression, somatic symptoms, anxiety, pain catastrophizing, perceived stress, jaw function, and sleep disturbance [37].

The Patient Health Questionnaire (PHQ) is a diagnostic tool for mental health disorders used by health care professionals. Studies have found good correlation between PHQ diagnoses and those of independent mental health professionals. The PHQ-9 includes nine symptoms of depression and assesses the level of the depression by the frequency of the symptoms within the last two weeks. Scores range between 0 to 27, and scores of 5, 10, 15, and 20 are considered cut-off values for mild, moderate, moderately severe, and severe depression, respectively. The PHQ-15 includes 15 somatic symptoms or symptom clusters that account for more than 90% of the physical complaints. Scores range between 0 to 30, and scores of 5, 10, and 15 are considered cut-off values for mild, moderate, and severe somatic symptoms, respectively [37, 38].

The Generalized Anxiety Disorder scale (GAD-7) assess generalized anxiety disorder symptoms and measures anxiety based on seven items. Total score ranges from 0 to 21, and scores of 5, 10, and 15 are considered cut-off values for mild, moderate, and severe anxiety, respectively [37].

The Perceived Stress Scale (PSS-10) assesses how unpredictable, uncontrollable, and overloaded individuals find their lives during the previous month. Total score ranges from 0 to 40, where a higher score indicates greater perception of stress [39]. In some studies, high PSS scores have been correlated to high biomarker levels of stress, such as cortisol [4, 40].

Pain Catastrophizing Scale (PCS) measures catastrophizing in the context of actual or anticipated pain. Total score ranges between 0 to 52, where higher scores indicate higher presence of catastrophizing thoughts. Previous studies have reported that a cut-off value of more than 30 points is associated with pain catastrophizing of clinical relevance [41].

Jaw Functional Limitation Scale (JFLS) assesses the function of the masticatory system in three dimensions: mastication, vertical jaw mobility, and emotional and verbal expression. The scale consists of 20 items with each item is rated with NRS, where 0 corresponds to no limitation and 10 to severe limitation. Calculation of a global score (0–10) as the average of the ratings for eight of the items is recommended [37], a higher score indicates insufficient jaw function [37].

Insomnia Severity Index (ISI) is a short screening instrument used to measure the symptoms of insomnia. The scale score range between 0 to 28, with a score above 15 indicating clinical insomnia [37].

### Subjective and semi-objective pain measures

#### Pain rating

All participants assessed their current pain intensity on the day of sample collection on a NRS. The scale ranges from 0 to 10, where 0 indicates “no pain at all” and 10 indicates “worst imaginable pain”.

Graded Chronic Pain Scale (GCPS) was used to assess pain intensity and pain-related disability. Subscales scores for pain intensity and disability are combined to enable classification of chronic pain in grade 0 (no pain) to IV (high disability-severe limiting).

The characteristic pain intensity (CPI) was also assessed (NRS) with the first three question of the GCPS. The pain intensity was calculated as the mean of the current pain intensity, and the average and worst pain intensity during the past month. The score was then multiplied by 10 to yield a 0–100 final score [37].

#### Pressure pain threshold

The pressure pain threshold (PPT) was assessed by an electronic pressure algometer (Somedic Sales AB, Hörby, Sweden). The device consists of a pistol grip and a rod with a pressure-sensitive strain gauge at the tip and a display unit. A circular padded probe with an area of 1 cm^2^ was used with increase of pressure rate of 50 kPa/s.

The PPT was recorded at the most prominent point of the masseter muscle, and over a reference point on the tip of the index finger on the same side. The most dominate side was used. The participants were instructed to press a hand-held button as soon as the pressure turned into a painful sensation, whereby the pressure value was frozen on a digital display. The procedure was first verbally described and illustrated for the participant. The PPT was then recorded three times at each location. For analyses, the average threshold of the three recordings was used.

### Sample collection

The participants were instructed not to eat, drink or brush their teeth during minimum one hour prior to sample collection. All samples were collected in the morning between 7:30 and 12:00 am to reduce the influence of diurnal variation, with more than 80% collected between 9:00–11:00 am.

Stimulated whole saliva was collected as earlier described by Jasim et al., 2016 and 2018 based on its simplicity, low variability, and significantly higher expression of several biomarkers [34, 42]. Prior to saliva collection, participants were instructed to rinse their mouth with water to remove any debris. Saliva samples were collected using paraffin gum (Orion Diagnostica, Finland). For pre-stimulation, the participants were instructed to chew the gum until it was smooth and homogenous. After 60 s of pre-stimulation, the participants were asked to swallow the saliva present in the mouth and then started to chew and expectorate the saliva into precooled polypropylene tube coated with protease inhibitor (Sigma Aldrich v/v 1:500) until 5 ml of whole stimulated saliva was achieved. The total spitting time was documented, and salivary flow was calculated.

Directly after saliva collection, a venous blood sample was collected in a 8.5 ml BD_TM_ P100 tube (BD, Franklin Lakes, New Jersey, US). The saliva and blood samples were gently mixed and directly centrifuged at 2500 x g for 15 min. The saliva supernatant and the plasma were alliquoted in 0.5 mL eppendorph vials and stored at − 70 °C until analysis.

### Chemical analyses

#### Glutamate

The concentration of glutamate in saliva and plasma was determined as described previously by Jasim et al. 2018 [42]. Briefly, 50 μl of the sample was centrifuged at 4 °C for 5 min at 12000 x g. The supernatant was collected, and 5 μl was immediately analysed using an ISCUSS Analyser (CMA Microdialysis, Solna, Sweden). The detection limit was 1.0 to 150 μmol/L.

#### Nerve growth factor and brain-derived nerve growth factor

Plasma and saliva samples were thawed on the day of analysis, blinded and randomly analysed for NGF (the active form *β*NGF was measured) and BDNF using multiplex electrochemiluminescence assay panel from Meso Scale Discovery (MSD, Rockville, MD, USA) according to the manufacturer’s protocol. Data were collected and analysed using MESO QUICKPLEX SQ 120 instrument (Meso Scale Diagnostics (MSD), Rockville, MD, USA) equipped with DISCOVERY WORKBENCH® data analysis software (MSD, Rockville, MD, USA). The limits of detection (LOD) were 0.036 pg/ml for NGF and 0.373 pg/ml for BDNF.

#### Substance P and serotonin

For detection of SP the enzyme-linked immunosorbent assay kit (ADI-900-018), and for 5-HT the colorimetric competitive enzyme immunoassay kit (ADI-900-175) from Enzo Life Sciences (Farmingdale, NY, USA) were used. The LOD for SP was 8.04 pg/ml and for 5-HT 0.293 ng/ml. Both kits were used according to the manufacturer instructions using 96 well plate. The plates were analyzed using a spectrophotometer (CLARIOstar®, BMG Labtech, Ortenberg, Germany).

### Statistics

Statistical analyses were performed using Statistica version 13 (StatSoft, Oklahoma, USA). The Shapiro-Wiks test was used to test for normality for each distribution. Salivary and plasma SP were normally distributed, while salivary and plasma NGF, BDNF, 5-HT and salivary glutamate was normally distributed after logarithmic transformation. Only plasma glutamate was not normally distributed. Substances that were detected in more than half of the samples were included in the statistical analysis. All samples were within the detection limit, only three saliva samples expressed levels of NGF and 5-HT slightly below the detection limit.

For continuous variables with normal distribution independent t-test was used. For categorical variable or variables that were non-normal distributed, Mann-Whitney U-test was applied to study differences between groups. To test for significant correlations between saliva and plasma levels of normally distributed mediators as well as PPT, the Pearson’s correlation test was used. Otherwise, correlations between variables were tested for statistical significance with Spearman correlation test. Correlations were adjusted for multiple comparisons according to Bonferroni. Descriptive data are presented as mean and standard deviation (SD) or median and interquartile range (IQR). For all analyses, the significance level was set at *P* < 0.05.

## Results

### Sample characterization

Descriptive data of patients and healthy controls are presented in Table 1. Patients and controls were similar in background factors, such as country of birth, occupation, education level, and level of physical activity.

**Table 1:** Descriptive data. Demographic features of patients with temporomandibular disorders myalgia (TMD) and healthy controls. Questionnaire scores are presented as mean ± standard deviation or as median (interquartile range). Statistical analyses were performed with independent t-test or Mann–Whitney U-test, *P* < 0.05)

Variable	TMD (*n* = 39)	Controls (*n* = 39)	Statistics
Body Mass Index (kg/m^2^)	23.7 ± 3.9	22.7 ± 3.3	NS
Age (Years)	28.8 ± 7.4	28.8 ± 6.9	NS
Sex (*n*, F/M)	32/7	32/7	NS
Body Mass Index (kg/m^2^)	23.7 ± 3.9	22.7 ± 3.3	NS
Number of teeth	28 (3)	30 (3)	NS
Pain-free opening (mm)	40.6 ± 9.9	56.5 ± 6.2	P < 0.001
Maximum unassisted opening (mm)	52.5 ± 6.4	57.9 ± 6.2	P < 0.001
Salivary Flow (ml/min)	1.6 ± 0.6	2.0 ± 0.9	*P* = 0.033
Pain duration (years)	6.7 ± 6.3	NA	*P* < 0.001
Current pain intensity (NRS)	4 (2)	0 (0)	P < 0.001
CPI	60 (20)	0 (0)	P < 0.001
PHQ-9 Score (0–36)	6 (7)	1 (4)	P < 0.001
PHQ-15 Score (0–30)	10 (7)	3 (4)	P < 0.001
GAD-7 Score (0–28)	4 (5)	1 (3)	P < 0.001
PSS-10 Score (0–40)	17 (11)	10 (9)	P < 0.001
JFLS Score (0–10)	1.2 (1.8)	0 (0)	P < 0.001
PCS Score (0–54)	14 (17)	3 (10)	P < 0.001
ISI Score (0–28)	10 (9)	5 (5.5)	P < 0.001
PPT reference (kPa)	356 ± 121	439 ± 119	*P* = 0.004
PPT masseter muscle (kPa)	180 ± 56	268 ± 71	P < 0.001

*n* number of subjects, *NRS* Numeric Rating Scale, *CPI* Characteristic Pain Intensity, *PHQ* The Patient Health Questionnaire, *GAD* Generalized Anxiety Disorder, *PSS* perceived stress scale, *JFLS* Jaw Functional Limitation Scale, *PCS* Pain Catastrophizing Scale, *ISI* Insomnia Severity Index, *PPT* Pressure Pain Threshold

Patients showed significantly higher signs of psychological distress and decreased jaw movements compared to controls. Even if the levels were significantly higher in patients compared to controls, the patients expressed on average mild depressive symptoms and insomnia, moderate levels of somatic symptoms and perceived stress, and almost no clinically relevant pain catastrophizing (Table 1).

### Glutamate

**Fig. 1:**
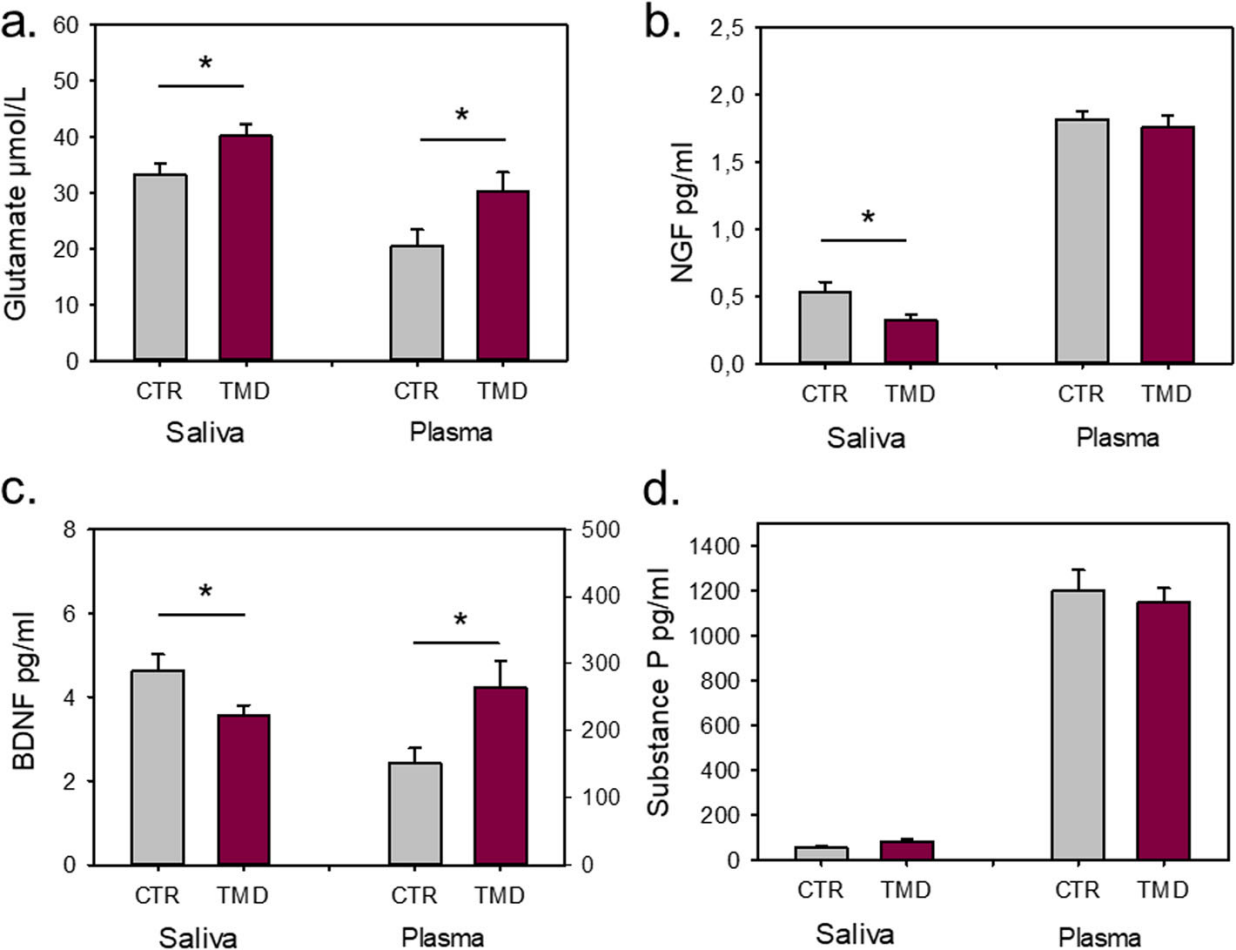
**a**-**d**. Salivary and plasma biomarkers. Salivary and plasma levels of glutamate, nerve growth factor (βNGF), brain derived neurotrophic factor (BDNF), and substance P (SP) in 39 patients diagnosed with temporomandibular myalgia according to the diagnostic criteria for temporomandibular disorders (TMD) and 39 healthy pain-free controls (CTR)

Salivary (t = 2.281; *n* = 66; *p* = 0.026) and plasma (Z = 2.03; *n* = 59; *p* = 0.043) levels of glutamate showed significant differences between patients and controls. Patients had significantly higher levels of glutamate both in saliva and in plasma compared to controls (Fig. 1a). Male TMD patients had higher levels of salivary glutamate compared to female patients (t = − 3.022; = 36; *p* = 0.005), but there were no sex differences in plasma levels.

There were no correlations between saliva and plasma glutamate levels.

### Serotonin

5-HT was poorly detected in saliva samples (36%) and was consequently removed from further analysis. Plasma 5-HT could be detected in 66% of all the blood samples There were no significant differences in plasma 5-HT levels between the two groups (t = − 0.907; *n* = 47; *p* = 0.370).

### Nerve growth factor

The levels of NGF in saliva and plasma are shown in Fig. 1b. Patients expressed significantly lower levels of salivary NGF in comparison to controls (t = − 2.194; *n* = 67; *p* = 0.032). A similar pattern with lower levels of NGF in patients compared to controls was found in plasma but the difference was not statistically significant (t = − 0.500; *n* = 69; *p* = 0.618). There were no sex differences in saliva and plasma NGF levels.

No correlation existed between the saliva and plasma levels of NGF.

### Brain-derived neurotrophic factor

BDNF expression differed significantly between groups in both saliva and in plasma, as reported in Fig. 1c. Salivary BDNF was lower in patients than in controls (t = − 2.247; *n* = 77; *p* = 0.028). Contrary, plasma BDNF was higher in patients compared to controls (t = 2.338; *n* = 68; *p* = 0.022). There were no sex differences in saliva and plasma BDNF levels.

There was no significant correlation between salivary and plasma BDNF levels.

### Substance P

SP was detected only in 52% of all the saliva samples but in almost all blood samples. The levels of SP in saliva and plasma are shown in Fig. 1d. Patients expressed slightly higher levels of SP in saliva compared to controls, but the difference was not significant (t = 1.771; *n* = 41; *p* = 0.084). The levels of SP in plasma were similar in both groups, without significant difference (t = − 0.458; *n* = 68; *p* = 0.649). There were no sex differences, and no significant correlation was found between salivary and plasma SP levels.

### Correlations

Table 2 shows the correlation coefficients (r or r_s_) between salivary levels of the substances and other variables among the two groups. There were no significant correlations for any substance among the patients, but BDNF showed moderate negative correlations to perceived stress, anxiety, and somatic symptoms among the healthy controls.

**Table 2:**
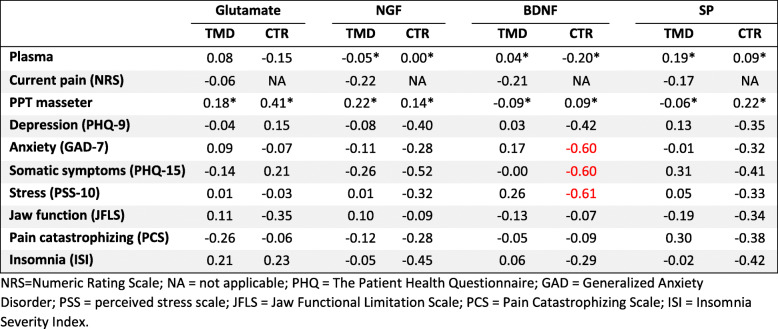
Correlation coefficient matrix. Overview of correlations for salivary glutamate, nerve growth factor (βNGF), brain derived neurotrophic factor (BDNF), and substance P (SP) in patients with temporomandibular disorders myalgia (TMD) and healthy controls (CTR). All correlations have been adjusted for multiple comparisons according to Bonferroni. Statistical analyses were performed with Pearson or Spearman* correlations test. Red marked correlations denote significance

In the groups combined, there was a moderate reverse correlation between NGF and somatic symptoms (r_s_ = − 0.462; *n* = 78; *p* < 0.001).

## Discussion

In this case-control study saliva and plasma levels of pain related biomarkers in patients with a diagnosis of TMD-myalgia and healthy controls were analyzed. The main findings were significantly different levels of salivary glutamate, NGF, and BDNF in patients with a diagnosis of TMD-myalgia compared to pain-free healthy controls. The patients also showed elevated plasma levels of glutamate and BDNF. However, there were no correlations to pain levels for any of the biomarkers, or between saliva and plasma levels. Thus, the results only partly support our hypothesis that saliva levels of certain well-known algesic substances reflect TMD pathophysiology.

The role of glutamate has previously been investigated in TMD myalgia. For example, intramuscular injections of glutamate into the masseter muscle of healthy individuals evoke pain and allodynia, and the volunteers reported similar discomfort as patients with TMD-myalgia [11]. Further, interstitial muscle levels of glutamate were elevated in TMD-myalgia compared to pain-free individuals [10, 13] and biopsies from human masseter muscles have revealed the presence of peripheral N-methyl-D-aspartate (NMDA) receptors [16]. Based on the close vicinity of the salivary glands and the masticatory muscles, we had hypothesized that glutamate (and other biomarkers) may diffuse into the saliva and that saliva levels thereby would mirror muscle levels. Our findings are also in accordance with a recent study showing higher levels of salivary glutamate in patients with chronic migraine compared to both episodic migraine and healthy controls [12]. However, the results regarding plasma glutamate contrasts a previous study that did not find any difference in plasma glutamate between TMD myalgia and pain-free controls [10]. The inconsistent results may be due to methodological and diagnostic dissimilarities, and the low number of subjects in the previous study making the results less reliable. There was however no correlation to pain level or psychological factors which is a draw back for the use of circulating glutamate as a biomarker of TMD myalgia. The higher levels of glutamate in male saliva may be a consequence of the decreased salivary flow rate in female patients compared to the males. We have previously shown that salivary glutamate levels increased with the flow rate [42].

5-HT could be detected in one third of the saliva samples and was therefore not further analyzed. As saliva is an ultrafiltrate of (platelet-poor) plasma, which contains only a very small portion of 5-HT (< 5%), a too low sensitivity of the immunoassay to detect 5-HT is a probable explanation. 5-HT is more expressed in platelet rich plasma compared to saliva; thus, it was easier to detect with the immunoassay and measurable levels were found in two thirds of the samples. The plasma levels were higher in healthy controls compared to patients, but within normal reference values [43, 44]. The non-significant differences in TMD-myalgia and healthy controls are in accordance with earlier studies in serum and plasma [44, 45].

The neuropeptide NGF regulates the development of the nervous system. The peptide has been recognized as a key mediator in chronic pain and has recently also been linked to depressive disorders [6, 46]. Based on previous studies of conditions associated with chronic pain [16–19, 21], elevated levels of NGF might be expected in TMD-myalgia. Contrary, in the current study we found significantly lower levels of salivary NGF in TMD-myalgia. However, these results are in line with a recent article of fibromyalgia [7] were plasma NGF were analysed. A possible explanation for the decreased levels of NGF in TMD-myalgia may be related to the psychological characteristics of these patients [7]. Individuals with TMD-myalgia, on average, exhibit greater psychological maladjustment compared to healthy controls [23]. They report significantly higher levels of depressive and somatic symptoms, psychosocial stress, anxiety, and pain catastrophizing than pain-free individuals [2–4, 6]. Patients in this study also scored these variables higher. Reduced levels of circulatory NGF have previously been reported in patients with depressive disorders [46]. Along with such interpretation, there was a moderate correlation between salivary NGF and somatic symptoms. Since decreased NGF has been associated with psychological impairment, our findings indicate that the decreased NGF levels in the study group may reflect comorbid psychological maladjustment usually associated with TMD myalgia and may not be related to the pain itself.

Several studies suggest the involvement of BDNF in pain processing and peripheral as well as central sensitization [26, 47, 48], why our finding of lower salivary BDNF in the patients was surprising. We have previously shown that BDNF levels are lower in stimulated whole saliva compared to resting whole saliva, and consequently the salivary BDNF are affected by the flow rate. One may therefore speculate if the lower levels in patients could be a consequence of the slightly lower flow rate (Table 1) [49]. Plasma BDNF levels on the other hand, showed as expected higher levels in TMD-myalgia. This finding is supported by previous studies in migraine and cluster headache [26] as well some other chronic pain conditions [7, 30, 48]. Therefore, the increase of plasma BDNF might be interpreted as a general reaction to nociception/pain [7], but this warrants further investigation.

The inverse correlation between salivary BDNF and perceived stress, anxiety and somatic symptoms among the controls are somehow in line with previous observations of decreased plasma/serum BDNF in mood disorders [50]. The lack of similar association in the patient group, which had higher levels of psychological distress indicate that the relation between BDNF and psychological distress may be more complex.

SP is an important mediator in pain perception and is involved in the transmission of pain from the periphery to the central nervous system and increased levels in patients with TMD-myalgia could therefore be expected. However, we found no differences in SP concentration between patients and controls. Previous studies on the salivary levels of SP in chronic pain are inconsistent. Some studies have shown decreased levels of SP in BMS and chronic low back pain compared to healthy pain-free controls [18, 23]. Contrary, another study reported increased SP levels in migraine and an association between high level of SP and high pain intensity [17]. A recent study demonstrated in similarity to our findings no differences in SP levels between patients with chronic neuropathic pain and healthy controls [22].

SP levels in plasma were similar compared to earlier studies [22, 42], while saliva showed lower levels [17, 23]. The latter difference is most probably due to different collection approaches. Previous studies have mostly analyzed SP in resting whole saliva, whereas in the current study stimulated whole saliva was collected. A recent study by our group established that stimulated whole saliva contained 20% of the levels detected in resting whole saliva [42]. This may also have affected the detectability in our saliva samples, resulting in measurable levels in 52% of the samples. Hence, for future studies it is recommended to use resting whole saliva for the detection of salivary SP.

Several substances enter the salivary gland from the blood by passing through the intercellular spaces by transcellular or paracellular diffusion, consequently saliva is often regarded as an ultrafiltrate of plasma and contains fractions of proteins derived from the blood stream [42, 49], which explains the usually lower concentration of NGF, BDNF and SP in saliva compared to plasma. Glutamate on the contrary showed higher levels in saliva, which imply a local secretion or even a diffusion from adjacent structures e.g. masseter muscle.

The present study has some limitations that need to be considered. The study was performed in adults between 18 and 40 years representing the peak of TMD prevalence; other age groups were not taken into consideration because of the possibility of age-variability [51]. Moreover, female participants represented a majority of the study population to mirror the distribution in the clinic where women in higher extent seek care for TMD-myalgia. Therefore, sex differences could not be properly addressed. Further, sex hormones can alter pain levels, and another limitation is that the female participants were not screened for menstrual cycle phase. However, it is most likely the women were in different phases of the menstrual cycle, which would remove such an effect. The immunohistochemistry analysis of 5-HT showed a major limitation because of the low concentration of the substance in saliva and plasma. Highly sensitive analytical methods such as mass spectrometry or high-performance liquid chromatography with electrochemical detection can be used in future study to be able to detect the low concentrations of 5-HT. Another limitation that may be further discussed is the influence of biomarkers related to stress e.g. cortisol. Stress is known to affect physiological functions and may interfere with putative biomarkers. We have in a previous study investigated salivary cortisol in TMD patients and found no differences between patients and healthy controls [4].

A strength of this study is that the diurnal variation of substances was taken into consideration, since all samples were collected in the morning hours with a majority of the samples collected between 09 and 11 am. The saliva collection was also standardized and followed a specific protocol in order to decrease the inter- and intra-individual variability. And finally, the study population were properly examined to exclude systemic or oral conditions that may affect the salivary composition and biomarker levels. Patients were also diagnosed by a clinician calibrated to a reference standard researcher (ME) according to the most recent DC/TMD criteria to ensure the diagnosis of TMD-myalgia.

## Conclusion

The present study showed that patients with a diagnosis of TMD-myalgia had significantly higher levels of salivary and plasma glutamate as well as plasma BDNF compared to healthy pain-free individuals, suggesting that these may be indicative biomarkers for TMD-myalgia. However, the levels did not significantly correlate to subjective pain levels, so the possibility for them to serve as biomarkers for TMD-myalgia can be questioned and warrants further investigations. Patients further showed lower levels of salivary NGF, which correlated to psychological maladjustment. This association may propose NGF as a possible marker for studying psychological dysfunction in TMD myalgia. Further studies are however needed to elucidate the mechanisms for NGF in TMD-myalgia.

## Data Availability

The datasets used during the current study are available from the corresponding author on reasonable request.

## Abbreviations

5HT: Serotonin
BDNF: Brain Derived Neurotropic Factor
BMS: Burning Mouth Syndrome
CPI: Characteristic Pain Intensity
DC/TMD: Diagnostic Criteria for Temporomandibular disorders
GAD: Generalized Anxiety Disorder
GCPS: Graded Chronic Pain Scale
JFLS: Jaw Functional Limitation Scale
ISI: Insomnia Severity Index
LOD: Limits of Detection
NGF: Nerve Growth Factor
NRS: Numeric Rating Scale
PCS: Pain Catastrophizing Scale
PHQ: Patient Health Questionnaire
PPT: Pressure Pain Threshold
PSS: Perceived Stress Scale
SP: Substance P
TMD: Temporomandibular Disorders
